# Nanodiamond Particles as Secondary Additive for Polyalphaolefin Oil Lubrication of Steel–Aluminium Contact

**DOI:** 10.3390/nano11061438

**Published:** 2021-05-29

**Authors:** Ankush Raina, Mir Irfan Ul Haq, Ankush Anand, Sanjay Mohan, Rajiv Kumar, Subramanian Jayalakshmi, Ramachandra Arvind Singh

**Affiliations:** 1 School of Mechanical Engineering, Shri Mata Vaishno Devi University, Katra 182320, Jammu and Kashmir, India; ankush.smvd@gmail.com (A.R.); haqmechanical@gmail.com (M.I.U.H.); anand.ankush13@gmail.com (A.A.); sanjaysmvdu@gmail.com (S.M.); rajivattri20@gmail.com (R.K.); 2 School of Mechanical and Electrical Engineering, Wenzhou University, Wenzhou 325035, China

**Keywords:** nanodiamond, PAO oil, copper oxide, hexagonal boron nitride, nanolubrication, wear, coefficient of friction

## Abstract

Nanodiamond (ND) particles are effective lubricant additives. Attention of research has shifted towards investigating the particles as secondary additives. ND particles provide more benefits as secondary additives than as the sole lubricant additive for steel–steel contacts. In this work, the influence of ND particles as secondary additives on oil lubrication of steel–aluminium tribopair (hard–soft contact) was examined. AISI 52100 steel balls were slid against AA2024 aluminium alloy discs, in the presence of polyalphaolefin (PAO) base oil, in boundary lubrication regime (applied normal load: 10 N to 50 N). Primary additives were copper oxide (CuO) and hexagonal boron nitride (h-BN) nanoparticles. The addition of ND particles to PAO, with CuO and h-BN as primary additives, at the lowest applied normal load of 10 N: (i) decreased the volumetric wear of the aluminium discs by 28% and 63%, respectively, and (ii) decreased the coefficient of friction by 15% and 33%, respectively. At the highest applied normal load of 50 N, it: (i) decreased the volumetric wear of the aluminium discs by 20% and 38%, respectively, and (ii) decreased the coefficient of friction by 5.4% and 8%, respectively. ND particles as secondary additives significantly reduce energy loss and power loss as a consequence of an effective reduction in friction during sliding. Unique characteristics of ND particles—such as their (a) physicochemical and thermal properties, (b) ball bearing and polishing effects and (c) synergistic interaction with primary additives to form stable tribofilms—enhance the lubrication performance of steel–aluminium contact. ND particles in combination with h-BN nanoparticles showed the best performance, due to better synergy between the primary additive and the secondary additive. Results from the investigation indicate that ND particles taken as secondary additives in small amount (0.2 wt%) can improve oil lubrication performance of hard–soft contacts in engineering systems.

## 1. Introduction

Nanodiamond (ND) particles are attractive as lubricant additives, especially as they are non-toxic and biocompatible. Due in part to these properties, ND particles are replacing lubricant additives, which have toxic elements of sulphur and phosphorous, owing to stringent environmental regulations [1]. A report by Mochalin et al. [2] gives a detailed account of the properties and applications of nanodiamonds. Recent work on commercial Cr cylinder liner/CrN-coated piston ring tests has identified the anti-scuffing ability of ND particles [3]. Diesel engine tests have shown that ND particles as engine oil additives increase maximum engine power and maximum torque, reduce entire engine’s mechanical loss, and enhance heat transfer and fuel efficiency [4]. Zhai et al. [5] have reported a reduction in friction and wear of fretting steel/copper interfaces due to the presence of ND particles in lubricant oil. Improvement in lubrication of AISI 52100 steel ball sliding against cast-iron diesel engine liner by SAE15W40 API CH-4 oil, upon addition of ND particles, has been observed by Javeed et al. [6]. ND particles have proved to improve lubrication performance of surfaces sliding under aqueous glycerol lubrication [7] and also for water lubrication [8]. Ivanov et al. [1] suggest that ND particles have potential as lubricant additives for engine oils, process lubricants, metalworking fluids, gear oils and hydraulic fluids.

ND particles as secondary additives, i.e., in combination with other primary additives, have shown increased benefits than when used as the sole additive to oils [1,9,10,11,12,13]. When ND particles were used in combination with zinc dialkyldithiophosphates (ZDDP) nanoparticles in 5W30 motor oil, it was reported that the amount of ZDDP required for improving antiwear performance reduced by five times due to synergism between ND particles and ZDDP nanoparticles [1]. ND particles, when used with molybdenum disulfide (MoS_2_)/tungsten disulfide (WS_2_) primary additives, resulted in effective lowering of friction coefficient and wear volume by two times when compared to those of the Poly alpha olefin (PAO) base oil [9]. The addition of ND particles to oil containing copper oxide (CuO) and hexagonal boron nitride (h-BN) nanoparticles reduced the coefficient of friction by 15.5% and 25.5%, respectively [10]. 5W30 Mobil oil with molybdenum dialkyldithiophosphate (MoDDP) nanoparticles, when incorporated with ND particles, showed reduction in coefficient of friction (COF) by 90% and reduction in wear scar area by more than a factor of two [11]. ND particles in combination with polytetrafluoroethylene (PTFE) particles reduced the coefficient of friction by 80% [13]. These studies [1,9,10,11,12,13] showed enhanced lubrication performance for steel–steel tribopairs (hard-hard contacts) under oil lubrication. ND particles as secondary additives for hard–soft contact has hitherto not been investigated. Such an investigation would provide insights for better lubrication of hard–soft tribopairs in engineering systems.

The present work examines the efficacy of utilizing ND particles as secondary lubricant additives for the steel–aluminium tribopair, i.e., for hard–soft contact. Automotive piston-cylinder assembly, aluminium/steel tool tribopair and aluminium-based bearings and steel shafts used in high-speed engines are some of the examples of hard–soft tribocontacts in engineering systems [14].

## 2. Experimental Details

### 2.1. Nanolubricants

**Figure 1:**
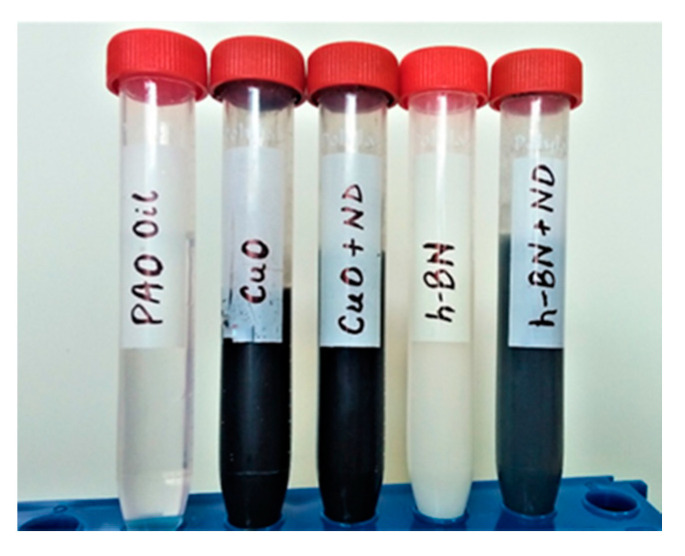
PAO base oil and four combinations of nanolubricants prepared, namely: (i) PAO + CuO, (ii) PAO + CuO + ND, (iii) PAO + h-BN and (iv) PAO + h-BN + ND.

Polyalphaolefin oil (PAO) was taken as the lubricant. Copper oxide (CuO) and hexagonal boron nitride (h-BN) nanoparticles were taken as primary additives and nanodiamond (ND) particles as secondary additives. The nanoparticles were procured from M/s Sigma–Aldrich (Bengaluru, India). Size (diameter) of the nanoparticles was in the range of 50 to 90 nm. Four combinations of nanolubricants were synthesized by suspending the nanoparticles in PAO base oil (Figure 1): (i) PAO + CuO, (ii) PAO + CuO + ND, (iii) PAO + h-BN and (iv) PAO + h-BN + ND.

Table 1 gives the rheological properties of the PAO base oil [10]. The nanolubricants were prepared in two steps. As the first step, 0.5 wt% CuO and 0.5 wt% h-BN nanoparticles were added to PAO base oil, separately (i.e., PAO + CuO and PAO + h-BN). In the next step, 0.2 wt% ND particles were added to the two nanolubricants prepared in the first step (i.e., PAO + CuO + ND and PAO + h-BN + ND). Concentration of nanoparticles was selected based on previous work [10]. Each nanolubricant combination was obtained by ultrasonication of nanoparticles in PAO base oil for 30 min using an ultrasonicator (Qsonica Sonicators, Newtown, CT, USA) to obtain uniform dispersions.

### 2.2. Tribological Testing

Tribological tests were conducted for steel–aluminium tribopair, with PAO base oil and the four nanolubricant combinations using a unidirectional ball-on-disc tribometer (DUCOM, Bengaluru, India). AISI 52100 100 Cr6 steel balls (radius: 5 mm, mean surface roughness: 0.020 ± 0.005 µm) were slid against aluminium alloy (AA2024-T6) discs (radius: 20 mm, thickness: 5 mm). The compositions of steel balls and aluminium alloy discs are given in Table 2. Surfaces of aluminium alloy discs were finished using emery papers of different grades (220, 400, 600, 800, 1000, 1500 and 2000), sequentially, prior to the tests. Mean surface roughness of the polished discs was 0.040 ± 0.005 µm, as measured using a 3D profilometer (Rtech Instruments, Cumming, GA, USA). Sliding tests were performed at five loads of 10, 20, 30, 40 and 50 N. Each test was conducted for 30 min, i.e., 900 cycles, corresponding to the sliding distance of 522 m. Test balls and discs were ultrasonically cleaned in acetone for 5 min before the tests. Friction force was continuously recorded during the tests with an in-built load cell (load range: up to 200 N). All tests were conducted at room temperature (25 ± 5 °C) and at the relative humidity of 40 ± 5%. Tests were repeated three times and the average values of coefficient of friction (COF) are reported.

Test parameters were chosen to ensure that lubrication occurred in boundary lubrication regime. Lambda (λ) value, the ratio of minimum film thickness to composite surface roughness, was calculated using Hamrock and Dowson’s formula (*h_min_*, Equation (1)) [15]. Estimated λ values were <1 (Table 3), indicating that the tribological tests were conducted in boundary lubrication regime.

(1) $$h_{min}=7.43R\left(1-0.85e^{-0.31k}\right){\left(\eta u/E^{*}R\right)}^{0.65}{\left(L/E^{*}R^{2}\right)}^{-0.21},$$

where *k* = ellipticity parameter (1), *η* = absolute viscosity (0.014 Pa·s), *u* = sliding velocity (0.29 m/s), *R* = composite radius (5 × 10^−^^3^ m), *L* = applied load (N), E* = composite elastic modulus (120.94 GPa). The estimated composite surface roughness was 0.044 μm.

### 2.3. Surface Analysis

Wear (volumetric loss) of the aluminium (Al) alloy discs was calculated as per ASTM G99 standard (Equation (2)) [16].

disc volume loss = π (wear track radius) (track width)/6 (ball radius)(2)

This approximate geometric relation is correct to 1% for (wear track width/ball radius) < 0.3, and is correct to 5% for (wear track width/ball radius) < 0.8 [16]. In the present case, the (wear track width/ball radius) is 0.18, for the largest wear track width. Wear track width was measured using an inverted metallurgical microscope (RMM-1, Jenoptik, Jena, Germany). Each reported value of wear (volumetric loss) is the average of values obtained from three tests. Worn surfaces of aluminium alloy discs were examined using a scanning electron microscope (SEM, JEOL, Akishima, Tokyo, Japan). Elemental composition of worn surfaces of discs was identified by energy dispersive spectroscopy (EDS). Microhardness of unworn Al alloy disc surface and that of the wear track of the Al alloy disc slid under PAO + h-BN + ND nanolubricant (50 N) was measured using Vicker’s microhardness tester (HVD 1000 M, Daksh Quality, Indore, MP, India), using a Vicker’s indenter (applied load: 5 N, dwell time: 10 s). The reported microhardness values are the average of values taken from 10 measurements.

## 3. Results and Discussion

### 3.1. Wear Behavior

**Figure 2:**
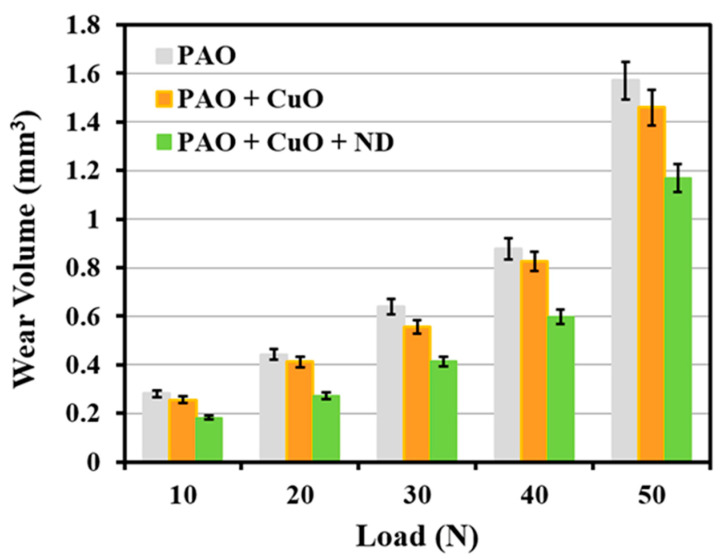
Wear volume of Al alloy discs (mm^3^) in the presence of PAO base oil, PAO + CuO and PAO + CuO + ND nanolubricants, with an increase in applied load from 10 N to 50 N.

**Figure 3:**
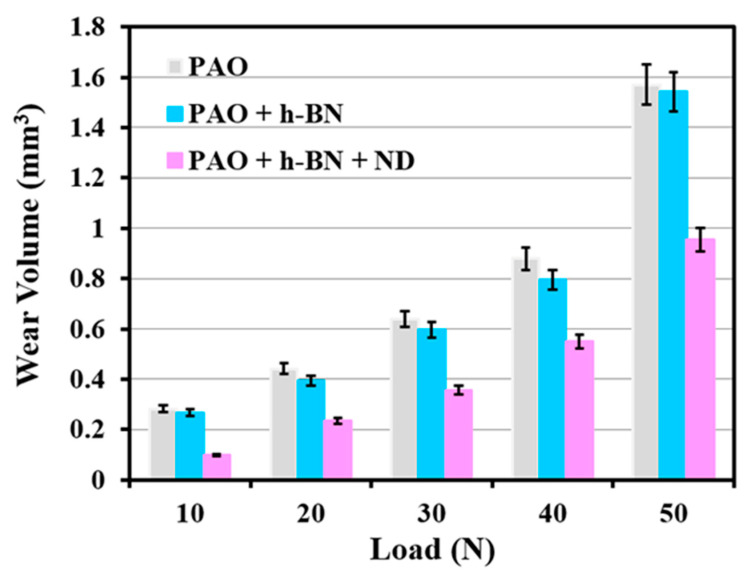
Wear volume of Al alloy discs (mm^3^) in the presence of PAO base oil, PAO + h-BN and PAO + h-BN + ND nanolubricants, with increase in applied load from 10 N to 50 N.

Volumetric wear (mm^3^) of Al alloy discs under lubrication by PAO base oil and the four nanolubricants as a function of applied normal load is shown in Figure 2 and Figure 3. Wear is highest at all the loads under PAO base oil lubrication, and it increases with the increase in load. When λ value is <1, as in the present case, oil film thickness is very small. In this regime, contact between asperities of tribopair takes place [17]. The trend of wear in the present case follows Archard’s wear model (wear in volume is proportional to load and to sliding distance, and inversely proportional to material hardness [18]). As load increases, the liquid film thickness (*h*_min_) becomes smaller (Table 3), which causes an increase in metal-to-metal contact, and consequently increases wear due to an increase in real contact area.

**Figure 4:**
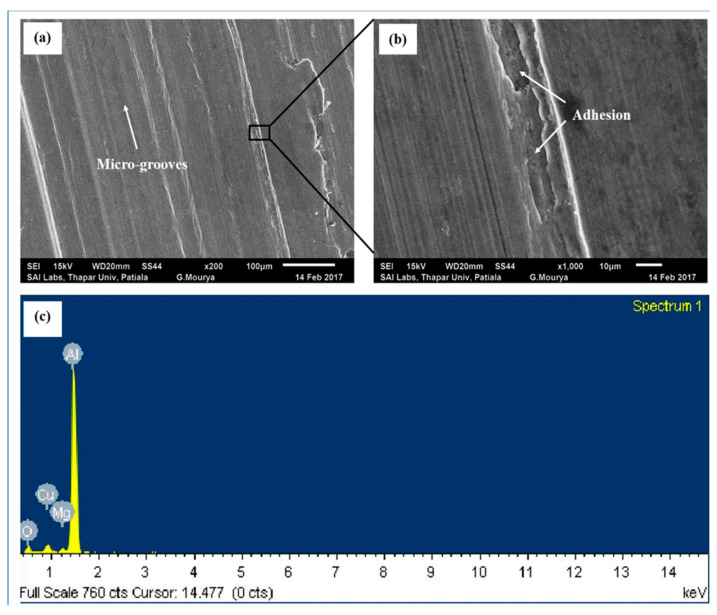
(**a**) SEM image of worn surface of the aluminium alloy disc lubricated with PAO base oil at 50 N load (200×); (**b**) magnified image (1000×) of Figure 4a; and (**c**) EDS spectrum of Figure 4a. Worn surface shows micro-grooves and occurrence of adhesion.

Worn surface of the disc under PAO base oil lubrication at the load of 50 N is shown in Figure 4a. Micro-grooves over the disc surface are visible, which are caused by the repeated sliding of hard asperities of steel counterface ball. The higher magnification image shows the occurrence of adhesion pits (Figure 4b). Material removal on the disc by abrasion of hard asperities of steel ball (i.e., indicated by the presence of micro-grooves) and adhesion cause considerable wear of the disc surface. Bai et al. [19] also observed that for steel–aluminium tribopair under lubrication, wear of aluminium occurs by abrasion and adhesion. EDS spectrum (Figure 4c) of the worn region (Figure 4a) shows elements Al, Cu and Mg of the aluminium alloy.

**Figure 5:**
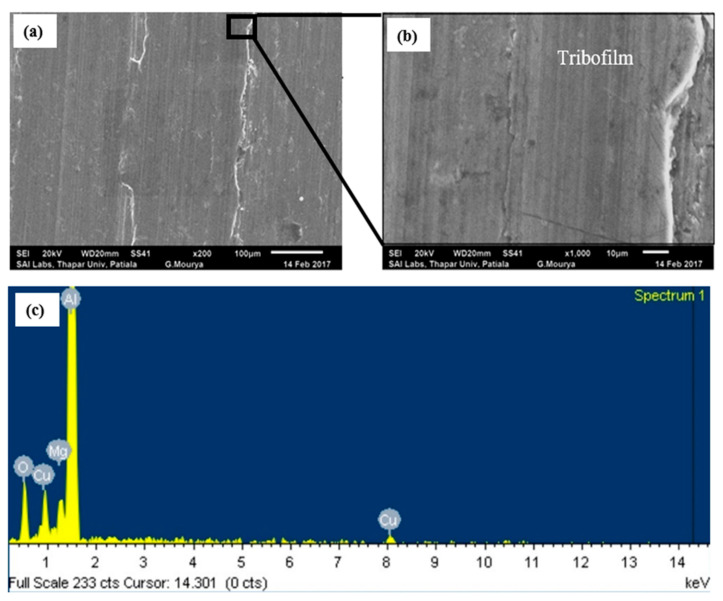
(**a**) SEM image of worn surface of the aluminium alloy disc lubricated with PAO + CuO nanolubricant at 50 N load (200×); (**b**) magnified image (1000×) of Figure 5a; and (**c**) EDS spectrum of Figure 5a. Worn surface is relatively smooth with tribofilm formation.

Under PAO + CuO lubrication, wear of Al alloy disc reduces when compared to that under lubrication by PAO base oil at all loads (Figure 2). The worn surface of the disc under PAO + CuO lubrication at the load of 50 N is shown in Figure 5a. Micro-grooves are absent and the worn surface appears relatively smooth when compared to that of the worn surface of the disc under PAO base oil lubrication (Figure 4a). A higher magnification image shows CuO tribofilm formation (Figure 5b). Tribofilm formation is a characteristic feature of CuO nanoparticles under oil lubrication [20]. Battez et al. [21] and Alves et al. [22] suggest that tribo-sintering of CuO nanoparticles under lubrication results in the formation of tribofilm, and consequently enhances tribological performance. CuO nanoparticles have load-bearing capacity [23] and form stable tribofilm, which prevents direct metal-to-metal contact between the tribopair, and reduces the wear of Al alloy disc.

**Figure 6:**
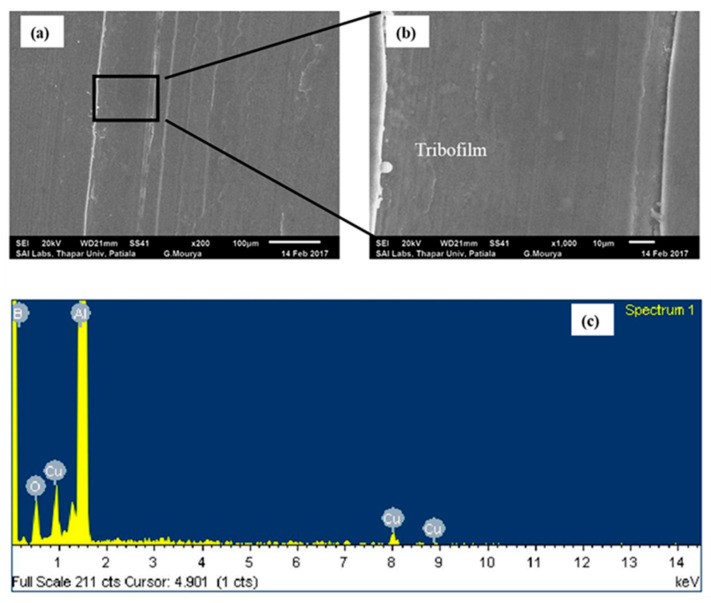
(**a**) SEM image of worn surface of the aluminium alloy disc lubricated with PAO + h-BN nanolubricant at 50 N load (200×) (**b**) magnified image (1000×) of Figure 6a; and (**c**) EDS spectrum of Figure 6a. Worn surface is smooth with tribofilm formation. Boron (B) peak in the EDS spectrum indicates the presence of h-BN nanoparticles on the worn surface.

Under PAO + h-BN lubrication, wear of Al alloy disc reduces when compared to that under lubrication by PAO base oil at all loads (Figure 3). The worn surface of the disc under PAO + h-BN lubrication at the load of 50 N is shown in Figure 6a. The worn surface is smooth with h-BN tribofilm formation, as can be seen at higher magnification (Figure 6b). Boron (B) peak in the EDS spectrum indicates the presence of h-BN nanoparticles on the worn surface (Figure 6c). During sliding, h-BN nanoparticles undergo shearing at the interface and form h-BN tribofilm on the disc surface, which prevents direct metal-to-metal contact between the tribopair and reduces wear [14,24,25]. Formation of h-BN tribofilm under lubrication has been previously reported by Charoo et al. [14]. Using Raman spectroscopy, they found that h-BN nanoparticles formed a tribofilm on the worn surface of grey cast iron (G-4000 grade), when h-BN nanoparticles were used as an additive to SAE 20W50 lubricant. Rio et al. [24], using Raman spectroscopy, detected the formation of h-BN tribofilm on AISI 420 stainless steel surfaces, when slid against AISI 52100 steel balls under trimethylolpropane trioleate (TMPTO) lubricant oil containing h-BN nanoparticles. Celik et al. [25] used EDS to identify the presence of h-BN on the worn surfaces of AISI 4140 steel, which was slid against WC 6% Co balls under the lubrication of SAE10W engine oil with h-BN nanoparticles. They reported that the presence of h-BN on the worn surface of AISI 4140 steel prevented direct contact with the sliding counterface balls, which consequently decreased friction and wear.

**Figure 7:**
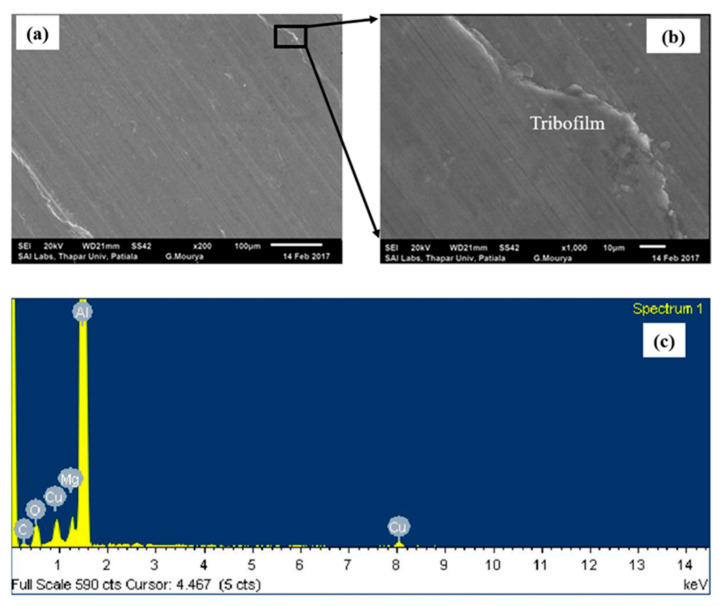
(**a**) SEM image of worn surface of the aluminium alloy disc lubricated with PAO + CuO + ND nanolubricant at 50 N load (200×) (**b**) magnified image (1000×) of Figure 7a; and (**c**) EDS spectrum of Figure 7a. Tribofilm formation can be seen on the worn surface. In the EDS spectrum, carbon (C) peak indicates the presence of ND particles on the worn surface.

Addition of ND particles to PAO + CuO nanolubricant (i.e., PAO + CuO + ND) decreases wear volume of Al alloy disc when compared to that under lubrication by PAO + CuO at all loads (Figure 2). At the lowest applied load of 10 N, a reduction of 28% in wear volume is observed due to the addition of ND particles to the PAO + CuO nanolubricant, and at the highest load of 50 N, there is a reduction of 20%. The worn surface of the disc tested with PAO + CuO + ND nanolubricant at the load of 50 N is shown in Figure 7a. Higher magnification image shows that the worn surface is smooth and is covered with CuO tribofilm (Figure 7b). Carbon (C) peak in the EDS spectrum indicates the presence of ND particles on the worn surface (Figure 7c).

**Figure 8:**
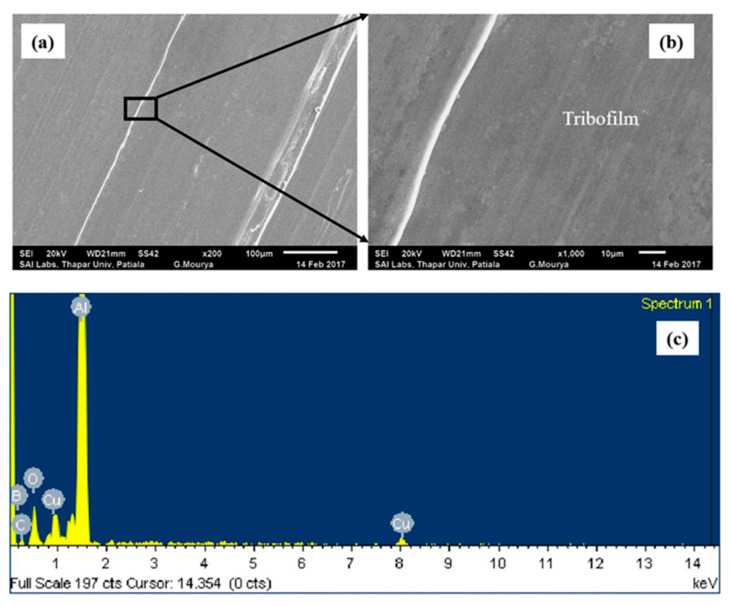
(**a**) SEM image of worn surface of the aluminium alloy disc lubricated with PAO + h-BN + ND nanolubricant at 50 N load (200×) (**b**) magnified image (1000×) of Figure 8a; and (**c**) EDS spectrum of Figure 8a. Tribofilm formation can be seen on the worn surface. In the EDS spectrum, boron (B) peak indicates the presence of h-BN nanoparticles and carbon (**c**) peak indicates the presence of ND particles on the worn surface.

The addition of ND particles to PAO + h-BN nanolubricant (i.e., PAO + h-BN + ND) decreases wear volume of Al alloy disc when compared to that under lubrication by PAO + h-BN at all loads (Figure 3). At the lowest applied load of 10 N, a reduction of 63% in wear volume is observed due to the addition of ND particles to the PAO + h-BN nanolubricant and at the highest applied load of 50 N, a reduction of 38%. The worn surface of the disc tested with PAO + h-BN + ND nanolubricant is shown in Figure 8a. The higher magnification image shows that the worn surface is smooth and is covered with h-BN tribofilm (Figure 8b). Boron (B) peak in the EDS spectrum indicates the presence of h-BN, and carbon (C) peak indicates the presence of ND particles on the worn surface (Figure 8c).

The average percentage of elements (wt%) present on the Al alloy disc surfaces slid under PAO base oil and four nanolubricants, from EDS area analysis, are given in Table 4. Compared to PAO base oil lubrication, in the case of PAO + CuO oil lubrication, (i) copper content is greater by 16.86% and oxygen content is greater by 12.04%, and (ii) aluminium content is lower by 28.36%. These results indicate the formation of CuO tribofilm on the surface slid under PAO + CuO nanolubrication. Compared to PAO base oil lubrication, in the case of PAO + CuO + ND oil lubrication, (i) copper content is greater by 14.52% and oxygen content is greater by 11.07%, (ii) aluminium content is lower by 32.15%, and (iii) carbon is present, at 7.34%. These results indicate the formation of CuO tribofilm, and the presence of ND particles on the surface and in the tribofilm formed on the surface slid under PAO + CuO + ND nanolubrication. Compared to PAO base oil lubrication, in the case of PAO + h-BN oil lubrication, (i) presence of boron (50.40%) and (ii) lower aluminium content (38.80%) indicate the formation of h-BN tribofilm on the surface slid under PAO + h-BN nanolubrication. Compared to PAO base oil lubrication, in the case of PAO + h-BN + ND oil lubrication, (i) presence of boron (51.06%), (ii) lower aluminium content (22.80%) and (iii) presence of carbon (18.62%) indicate the formation of h-BN tribofilm, and the presence of ND particles on the surface and in the tribofilm formed on the surface slid under PAO + h-BN + ND nanolubrication.

**Figure 9:**
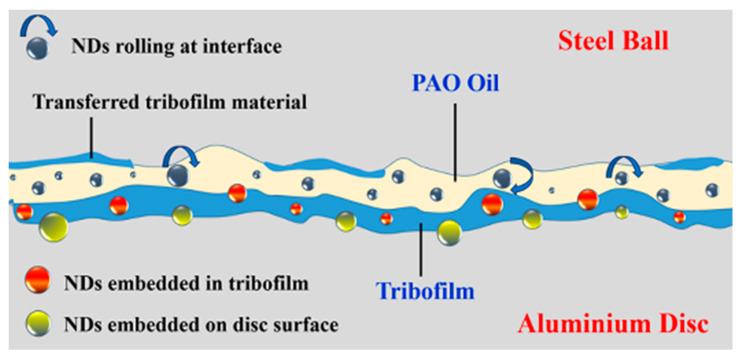
Schematic showing: (i) rolling of ND particles at interface (ball bearing effect); (ii) ND particles embedded in tribofilm; (iii) ND particles embedded on aluminium alloy disc surface; and (iv) transferred tribofilm material on steel ball surface (ND: nanodiamond).

**Figure 10:**
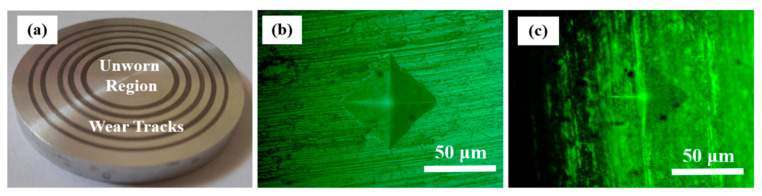
(**a**) Al alloy disc tested with PAO + h-BN + ND nanolubricant at 50 N. Microhardness indentation (**b**) on unworn region of Al alloy disc surface (hardness: 148 Hv), magnification 40× and (**c**) on the wear track of Al alloy disc tested with PAO + h-BN + ND nanolubricant at 50 N (hardness: 190 Hv), magnification 40×.

**Figure 11:**
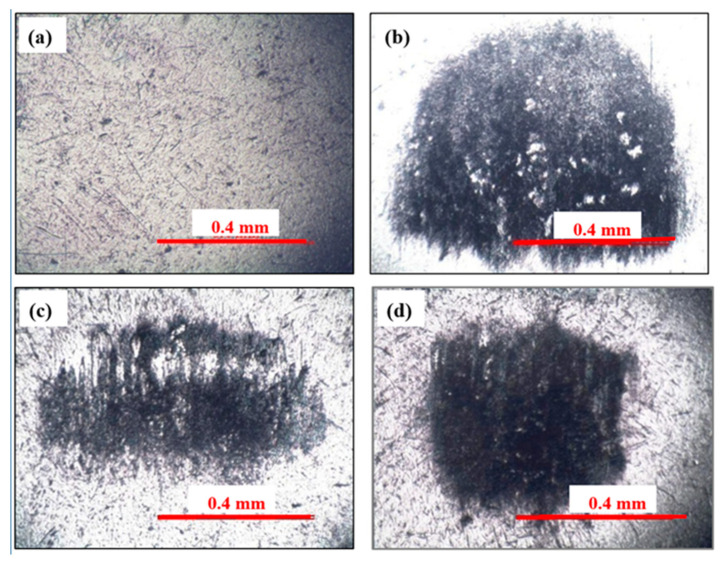
Optical microscope images of steel ball (**a**) unworn surface; and worn surfaces showing material transfer to steel balls tested (**b**) with PAO base oil, (**c**) with PAO + CuO + ND nanolubricant and (**d**) PAO + h-BN + ND nanolubricant.

ND particles polish the sharp asperities of sliding surfaces, in the absence of which sharp asperities would experience high stress causing high wear [1]. ND particles become embedded in CuO and h-BN tribofilms (Figure 9), which increases their load-bearing capacity and hardness, which, in turn, enhance protection of Al alloy discs against wear [1]. Microhardness of unworn Al alloy disc surface and that on the wear track of the Al alloy disc slid with PAO + h-BN + ND nanolubricant (50 N) was measured (Figure 10). Hardness value for the unworn surface was 148 Hv, whereas on the wear track of the disc slid with PAO + h-BN + ND nanolubricant, the value was 190 Hv, an increase of 28%. Optical images of the worn surfaces of steel balls tested with the PAO base oil, PAO + CuO + ND nanolubricant and PAO + h-BN+ ND nanolubricant at 50 N applied load are shown in Figure 11a–d. The wear scar is largest for the steel ball tested with the PAO base oil (Figure 11a). Lumps of Al alloy material picked up from the disc surface due to adhesion during sliding (Figure 4b) can be seen as the white areas within the wear scar. Transferred tribofilm material can be observed on the surfaces of the steel balls slid against Al alloy discs under PAO + CuO + ND and PAO + h-BN+ ND nanolubricants. The smearing of h-BN + ND material over the steel ball surface is prominent (Figure 11d), as h-BN is a soft material. Transfer of tribofilm material with ND particles to the surfaces of steel balls lowers the wear of Al alloy discs [1]. Compared to the worn surface of the Al alloy disc lubricated with PAO base oil (Figure 4), the morphology of the worn surfaces of the Al alloy discs lubricated by the four nanolubricants (Figure 5, Figure 6, Figure 7 and Figure 8) is drastically different. Their worn surfaces appear smoother and have finer grooves. The former is due the presence of tribofilms on the worn surfaces of the discs, and the latter is due to their rubbing against material transferred to the steel balls.

ND particles, being harder than Al alloy discs, become embedded on the disc surfaces (Figure 9). Embedded ND particles increase the surface hardness of Al alloy discs, and also mechanically lock the tribofilms and retain these films on the surfaces of discs (Figure 9), which ensures sustained surface protection. Embedment of ND particles on sliding surfaces has been identified by Novak et al. [26] using X-ray photoelectron spectroscopy (XPS) and by Alias et al. [27] using Raman spectroscopy. Liu et al. [4], upon conducting diesel engine tests with engine oil using ND particles as additives, found that ND particles due to their higher hardness penetrated the test surfaces. Tao et al. [28], in their work on lubrication of steel–steel tribopairs with paraffin oil with 1% ND particles, identified the presence of embedded ND particles on the slid surfaces using XPS. Hardness measurements revealed that, due to the embedment of ND particles on the sliding surfaces, the hardness value increased by 136 Hv. Puzyr et al. [29], in their investigation on the lubrication of steel–steel tribopairs with SAE 10W oil with 0.01 wt% ND particles, found that, post-test, the hardness of the rubbed surface increased by almost 1 GPa due to the embedment of ND particles on the metal surface.

At the highest load of 50 N, the wear coefficient (K) of Al alloy discs under lubrication by PAO base oil is 5.91 × 10^−5^, which is three orders lower than severe wear (typically for mild wear, K ≈ 10^−8^, whereas for severe wear, K ≈ 10^−2^ [18]). At loads higher than those used in the present work, Al alloy would experience severe wear, and at much higher loads it would fail catastrophically by scuffing [30]. ND particles would prove beneficial at higher loads in terms of lowering wear, as they have anti-scuffing ability [3].

### 3.2. Friction Behaviour

**Figure 12:**
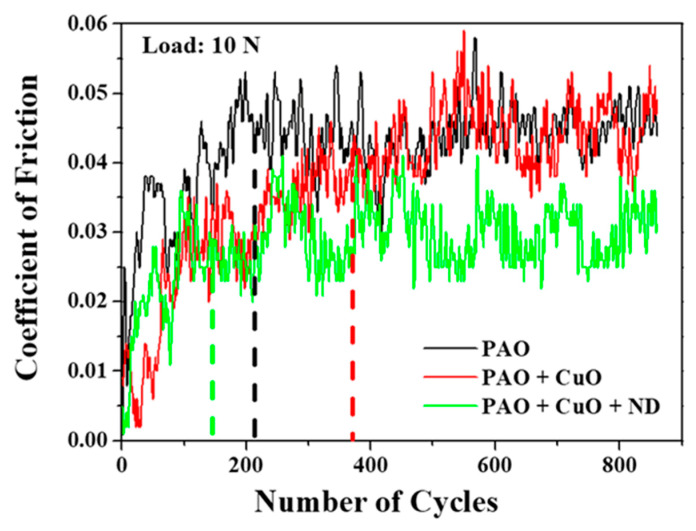
Coefficient of friction as a function of the number of cycles for PAO base oil, PAO + CuO and PAO + CuO + ND nanolubricants (tests conducted at 10 N load). The vertical dotted lines intersecting the *x*-axis indicate the onset of steady state condition.

**Figure 13:**
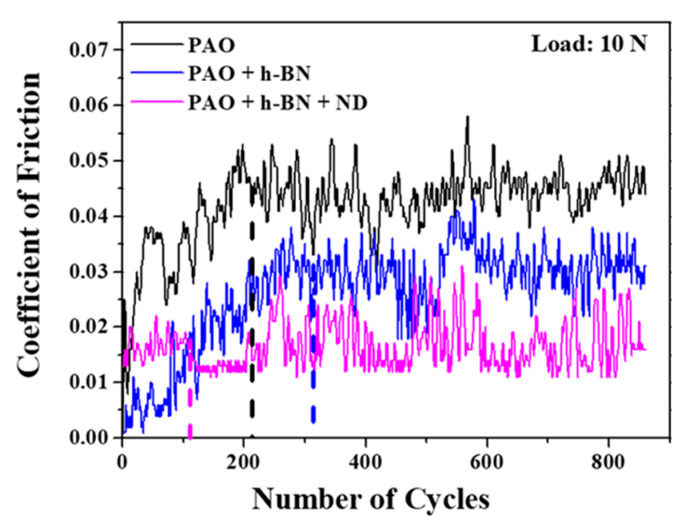
Coefficient of friction as a function of the number of cycles for PAO base oil, PAO + h-BN and PAO + h-BN + ND nanolubricants (tests conducted at 10 N load). The vertical dotted lines intersecting the *x*-axis indicate the onset of steady state condition.

Coefficient of friction (COF) traces of the tribopair as a function of the number of cycles in the presence of PAO base oil, PAO + CuO and PAO + CuO + ND nanolubricants at 10 N applied load are shown in Figure 12. COF for all lubricants increases with the increase in the sliding cycles, and eventually reaches a steady state value. Steady state is reached approximately at 210 cycles for PAO base oil, 370 cycles for PAO + CuO nanolubricant and at 140 cycles for PAO + CuO + ND nanolubricant. The steady state COF for the PAO base oil was in the range of 0.041 to 0.047 (average COF value 0.043). For PAO + CuO nanolubricant, the average COF value was 0.039, which is lower than that of the PAO base oil. For PAO + CuO + ND nanolubricant, the average COF value was 0.033, which is lower when compared to those of the PAO base oil and PAO + CuO nanolubricant. For PAO + CuO and PAO + CuO + ND nanolubricants, the average steady state COF values are in the order: PAO base oil (0.043) > PAO + CuO (0.039) > PAO + CuO + ND (0.033). COF traces of the tribopair as a function of number of cycles in the presence of PAO base oil, PAO + h-BN and PAO + h-BN + ND nanolubricants at 10 N applied load are shown in Figure 13. COF for all lubricants increases with the sliding cycles and reaches a steady state value. Steady state is reached approximately at 310 cycles for PAO + h-BN nanolubricant and at 110 cycles for PAO + h-BN + ND nanolubricant. The average COF value for PAO + h-BN nanolubricant was 0.030, which is lower than that of the PAO base oil. The addition of ND particles further reduced the average COF value to 0.017. For PAO + h-BN and PAO + h-BN + ND nanolubricants, the order is: PAO base oil (0.043) > PAO + h-BN (0.030) > PAO + h-BN + ND (0.017).

**Figure 14:**
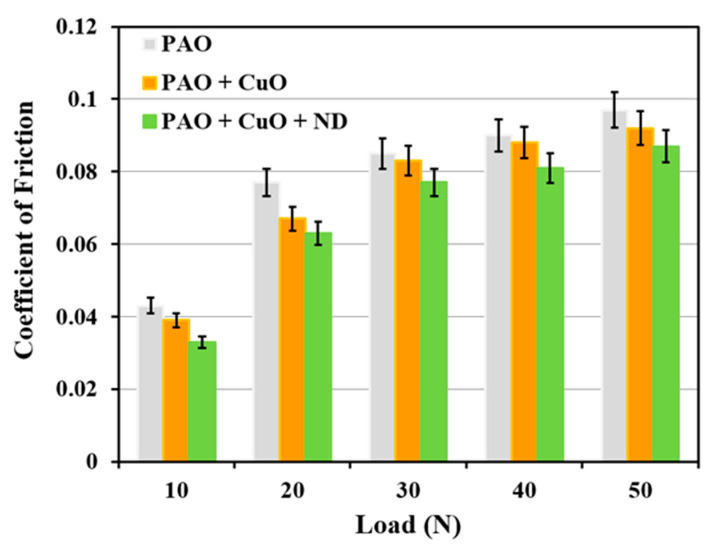
Coefficient of friction of the steel–aluminium tribopair with PAO base oil, PAO + CuO and PAO + CuO + ND nanolubricants, with increase in applied load from 10 N to 50 N.

Average steady state COF values of the tribopair in the presence of PAO base oil, PAO + CuO and PAO + CuO + ND nanolubricants for applied loads 10 N to 50 N are shown in Figure 14. Coefficient of friction is highest at all the loads under PAO base oil lubrication, and it increases with load. In boundary lubrication regime, as in the present case, film thickness is smaller than the composite surface roughness (Table 3), due to which friction is influenced by the physical interaction of both the solid surfaces. With an increase in load, asperity to asperity contact between sliding surfaces increases, which leads to an increase in the coefficient of friction. The occurrence of adhesion pits and the presence of micro-grooves on the worn surface of the Al alloy disc under PAO base oil lubrication (Figure 4) indicate that both the components of friction, namely, adhesive component (F_a_, Equation (3)) and ploughing component (F_p_, Equation (4)) [18] influence the coefficient of friction. Additionally, the coefficient of friction is also affected by the material transferred to the steel ball surfaces and wear debris (third body) at the interface.

F_a_ = τ A_r_, (3)

where τ is the shear strength, an interfacial property and A_r_ is the real area of contact.

F_p_ = d^3^P/12 R, (4)

where d is the track width, P the mean pressure required to displace the material in the surface and R the radius of curvature of the steel ball.

**Figure 15:**
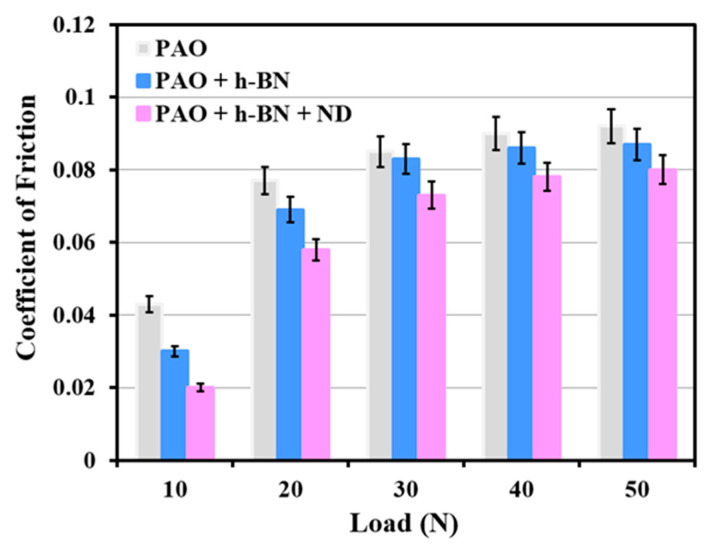
Coefficient of friction of steel–aluminium tribopair with PAO base oil, PAO + h-BN and PAO + h-BN + ND nanolubricants, with increase in applied load from 10 N to 50 N.

Under PAO + CuO lubrication, COF reduces when compared to that under lubrication by PAO base oil at all loads (Figure 14). During sliding, CuO nanoparticles form tribofilm on disc surface (Figure 5) and the tribofilm prevents direct metal-to-metal contact between the tribopair and reduces the friction property [10]. Average steady state COF values of the tribopair in the presence of PAO base oil, PAO + h-BN and PAO + h-BN + ND nanolubricants for applied loads 10 N to 50 N are shown in Figure 15. Under PAO + h-BN lubrication, COF reduces when compared to that under lubrication by PAO base oil at all loads. h-BN nanoparticles have a layered structure, similar to graphite, MoS_2_ and WS_2_ solid lubricants, with strong covalent bonds between their molecules in each layer and weak van der Waals bonds between the layers [14,24,25,31]. Under external shear force, such as that during sliding under an applied load, easy shearing takes place between the layers along the basal plane of h-BN structure, and the material becomes smeared onto the disc surface as a tribofilm (Figure 9). This occurrence causes a reduction in the COF [14,24,25].

The addition of ND particles to PAO + CuO nanolubricant (i.e., PAO + CuO + ND) decreases COF values when compared to that under lubrication by PAO + CuO at all loads (Figure 14), such that at the lowest applied load of 10 N, a reduction of 15% in COF is observed, and at the highest applied load of 50 N, there is a reduction of 5.4%. During the initial period of sliding, ND particles polish the sharp asperities of sliding surfaces and make them blunt, reducing COF (in the absence of polishing, rubbing of sharp asperities of sliding surfaces would cause high COF) [1]. The surface-polishing effect by ND particles in the initial period, i.e., running-in period of tribotests, has been reported in steel–steel tribopair lubricated with paraffin oil with 1% ND particles by Tao et al. [28], and similar observations have also been reported by other research groups [7,10,32]. The addition of ND particles to oil with molybdenum dialkyldithiophosphate (MoDDP) nanoparticles has shown improvement in surface finish, with surfaces becoming smoother by 35% [11]. Javeed et al. [6] in their investigation on SAE15W40 API CH-4 oil lubrication with ND particles of AISI 52100 steel ball sliding against cast-iron diesel engine liner, observed a 66% decrease in the roughness of the surface of the liner material, ascribed to the polishing effect of ND particles. Polished surfaces, i.e., smoother surfaces, provide better compliance for formation of uniform and conformal tribofilms [1]. ND particles are hard (Mohs hardness 10 [33]) and so they become embedded in the tribofilm of CuO nanoparticles, which are relatively soft (Mohs hardness 3.5 [23]). ND particles have an octagonal shape, i.e., an almost spherical shape [10], and, due to their shape, those particles that are free (i.e., un-embedded particles) undergo rolling. As the ball slides over the disc, ND particles undergo rolling at the interface of the tribopair, as illustrated in the schematic (Figure 9). The rolling of ND particles is similar to the rolling of balls between raceways in a bearing, and thus this mechanism is referred to as the “ball bearing effect” [10,28]. Tao et al. [28] opined that the rolling action of ND particles at sliding interfaces is a major mechanism that contributes towards the lowering of friction under oil lubrication of steel–steel tribopairs. Kim et al. [34] mentioned that the ball bearing effect of ND particles promotes lubrication. Novak et al. [26] reported that the rolling of ND particles was the mechanism for reduction in friction under oil lubrication. In the present case, the rolling effect of ND particles lowers COF during PAO + CuO + ND lubrication. The addition of ND particles to PAO + h-BN nanolubricant (i.e., PAO + h-BN + ND) decreases COF values when compared to that under lubrication by PAO + h-BN at all loads (Figure 15), such that at the lowest applied load of 10 N, a reduction of 33% in COF is observed, and at the highest applied load of 50 N, there is a reduction of 8%. During sliding, ND particles roll/slide at the interface, and, by this means, they promote shearing of h-BN nanoparticles and their increased smearing onto disc surfaces (Figure 9 and Figure 11d). Due to the rolling action of ND particles and the increased shearing of h-BN nanoparticles induced by ND particles, the friction property reduces significantly during PAO + h-BN + ND lubrication. Similar results have been found by Raina et al. [10], for sliding between steel–steel tribopairs.

### 3.3. Nanodiamond Particles as Secondary Additives

**Figure 16:**
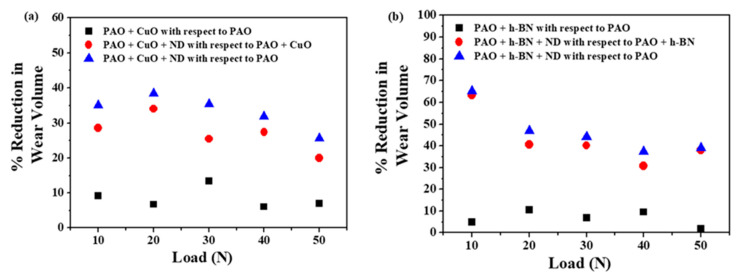
Percentage reduction in the average values of wear volume of Al alloy discs under: (**a**) PAO + CuO primary additive/+ ND secondary additive nanolubricants and (**b**) PAO + h-BN primary additive/+ ND secondary additive nanolubricants.

**Figure 17:**
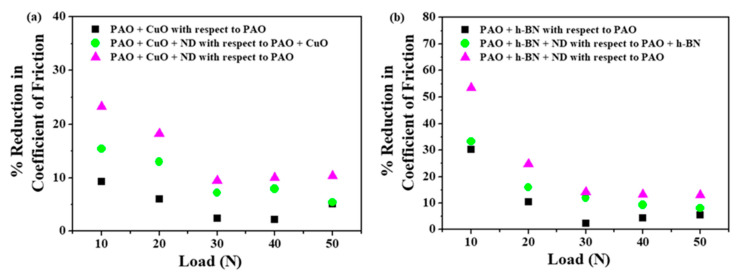
Percentage reduction in the average values of COF of steel–aluminium tribopairs under: (**a**) PAO + CuO primary additive/+ ND secondary additive nanolubricants and (**b**) PAO + h-BN primary additive/+ ND secondary additive nanolubricants.

The percentage reductions in the average values of the wear volume of Al alloy discs with nanolubricants at all loads are shown in Figure 16. The addition of ND particles to PAO + CuO and PAO + h-BN nanolubricants decreases wear volume at all applied loads. The reduction in wear volume is remarkable, even at the highest applied load (50 N), probably due to increased compaction of tribofilms, and also due to their transfer to the surface of steel balls (Figure 11) [1]. The percentage reductions in the average values of COF with nanolubricants at all loads is shown in Figure 17. The addition of ND particles to PAO + CuO and PAO + h-BN nanolubricants decreases COF values at all applied loads.

Energy loss due to sliding under lubrication with PAO base oil and the four nanolubricants at all loads is given in Table 5. Energy loss is found to be highest under lubrication by PAO base oil at all loads. At the lowest applied load of 10 N, PAO + CuO + ND nanolubricant reduces the energy loss by 31 J compared to that under PAO + CuO lubrication (i.e., a reduction of 15%), and at the highest applied load of 50 N, it reduces the energy loss by 131 J (i.e., a reduction of 5.4%). At the lowest load of 10 N, PAO + h-BN + ND nanolubricant reduces the energy loss by 52 J, compared to that under PAO + h-BN lubrication (i.e., a reduction of 33%), and at the highest load of 50 N, it reduces the energy loss by 182 J (i.e., a reduction of 8%).

Sliding of surfaces is a dynamic process, and, hence, power loss during sliding of the tribopair is an important factor that needs to be considered. Power loss under lubrication with PAO base oil and the four nanolubricants at all loads is given in Table 6. Power loss is found to be highest under lubrication by PAO base oil at all loads. At the lowest applied load of 10 N, PAO + CuO + ND nanolubricant reduces the power loss by 17 mW, compared to that under PAO + CuO lubrication (i.e., a reduction of 15%) and at the highest applied load of 50 N, it reduces the power loss by 73 mW (i.e., a reduction of 5.4%). At the lowest applied load of 10 N, PAO + h-BN + ND nanolubricant reduces the power loss by 29 mW, compared to that under PAO + h-BN lubrication (i.e., a reduction of 33%) and at the highest applied load of 50 N, it reduces the power loss by 101 mW (i.e., a reduction of 8%). The addition of ND particles as the secondary additives to the base oil with primary additives reduces energy loss and power loss significantly as a consequence of effective reduction in COF.

In light of the observations made in the present work and from earlier reports [1,9,10,11,12,13], the enhanced lubrication effect induced by ND particles as secondary additives arises due to their unique characteristics: (i) physicochemical and thermal properties; (ii) polishing and rolling effects; and (iii) synergistic interactions with primary additives. To elucidate: (i) ND particles are chemically and thermally stable. They have higher thermal conductivity (ND 1000-3300 W/mK, [35,36]) than the primary additives (CuO 30 W/mK [36], h-BN 550 W/mK (in-plane) and 5 W/mK (out-of-plane) [37]). Thermal conductivity of ND particles is four orders higher than that of PAO oils (0.143 W/mK [34,38]). Due to their high thermal conductivity and diminutive size (nanosized particles have large surface area to volume ratio), ND particles provide greater heat dissipation. Kim et al. [34] investigated lubrication performance of paraffin liquid containing ND particles for AISI 52100 bearing steel balls slid against AISI 1020 steel. The temperature of the balls was measured during the tests, and by these measurements it was found that the time to scuffing was delayed by ND particles, due to their high thermal conductivity, which effectively dissipated heat generated at the interface. Liu et al. [4] conducted diesel engine tests with engine oil using ND particles as additives. They reported that ND particles increased the thermal conductivity of the base oil, such that the thermal conductivity of the oil increased with the content of ND particles. The ND particles increased the overall heat transfer coefficient. By the addition of ND particles to engine oil, enhanced heat transfer and increased fuel efficiency was achieved. Friction energy includes strain energy, fracture energy and thermal energy [39]. Thermal energy induces plastic deformation, and, hence, effective dissipation of thermal energy, i.e., heat dissipation at the sliding interface suppresses plastic deformation of sliding surfaces. (ii) ND particles are hard, nanosized and are almost spherical in shape; consequently they polish the sliding surfaces and roll at the interface, reducing wear and friction (in contrast, micron-sized particles can increase wear and friction via a ploughing action). The smoothening of sliding surfaces due to the polishing effect promotes uniform and conformal tribofilm formation. (iii) ND particles are hard; thereby they have high load-bearing capacity, and reinforce tribofilms or relatively softer sliding surfaces, and impart higher hardness that lowers wear. Stable tribofilms obviate direct metal-to-metal contact, decreasing friction and wear. ND particles embedded on sliding surfaces can mechanically lock tribofilms and retain them on sliding surfaces, which promotes sustained surface protection. ND particles shear soft solid lubricants and reduce friction.

The polishing effect of ND particles reduces the running-in time, and steady state is reached at a lesser number of cycles (Figure 12 and Figure 13). At 10 N applied load, steady state is reached approximately at 210 cycles for PAO base oil, and for the nanolubricants at 370 cycles for PAO + CuO, 310 cycles for PAO + h-BN, 140 cycles for PAO + CuO + ND and 110 cycles for PAO + h-BN + ND nanolubricant. Delay in the onset of steady state is observed for the PAO base oil containing the primary additives (PAO + CuO and PAO + h-BN), which is probably due to the time required for the formation of tribofilms. The addition of ND particles to the PAO base oil containing the primary additives (PAO + CuO + ND and PAO + h-BN + ND) reduces the number of cycles for the onset of steady state, such that the number of cycles is lower than that for the PAO base oil. ND particles have good synergy with the primary additives (CuO and h-BN) and accelerate the formation of stable tribofilms.

ND particles effectively contribute towards the lowering of the COF of the steel–aluminium tribopair due to their: (a) physicochemical and thermal properties; (b) ball bearing and polishing effects; and (c) synergistic interaction with primary additives to form stable tribofilms. Reduction in energy loss and power loss, as the consequence, are indicative of enhanced lubrication of the tribopair. From the observations made in the present work and from earlier reports [1,9,10,11,12,13], the synergistic interactive mechanisms of ND particles with tribo-components are summarized in Table 7. Such synergetic interactions of ND particles with tribo-components provide greater benefits than when they are used as the sole additive to oils.

### 3.4. Comparative Performance of Combination of Lubricant Additives

Comparatively, PAO + h-BN + ND nanolubricant shows better lubrication performance than PAO + CuO + ND nanolubricant, in terms of wear and friction reduction (Figure 16 and Figure 17). The better performance of PAO + h-BN + ND nanolubricant is ascribed to (i) the inherent physicochemical and thermal properties of h-BN nanoparticles and (ii) the better synergistic interaction between ND particles and h-BN nanoparticles. To elucidate: (i) h-BN is the softest and most lubricious polymorph of BN [24,40], due to which the nanoparticles undergo easy shearing and reduce friction at the interface. h-BN nanoparticles have excellent chemical and thermal stability. Their thermal conductivity is higher, especially along the basal plane, the direction in which they shear easily (550 W/mK (in-plane) and 5 W/mK (out-of-plane) [35]), than CuO nanoparticles (30 W/mK [36]). Hence, they provide higher heat dissipation at the interface. The density of h-BN nanoparticles (2.3 g/cm^3^, [14]) is lower by 2.74 times than that of CuO nanoparticles (6.32 g/cm^3^, Sigma–Aldrich Material Safety Data Sheet). This means that for the same wt%, a greater amount of h-BN is available for lubrication. (ii) ND particles during their rolling/sliding motion cause increased shearing of h-BN nanoparticles, and their increased smearing onto disc surfaces. EDS analysis data (Table 4) show that on the Al alloy disc surface slid under PAO + h-BN + ND nanolubrication, (i) aluminium reduction rises by 33% and (ii) presence of carbon rises by 11%, when compared to that of the Al alloy disc surface slid under PAO + CuO + ND nanolubrication. These results indicate that the h-BN tribofilm with ND particles has larger area coverage over the wear track than that of the CuO tribofilm with ND particles. h-BN tribofilm with interspersed ND particles provides better protection to the sliding surfaces against wear, and also lowers friction.

COF of the tribopair is lower for the PAO + h-BN + ND nanolubricant than for the PAO + CuO + ND nanolubricant, and, as a direct consequence, the PAO + h-BN + ND nanolubricant causes lower energy loss and lower power loss during sliding compared to the PAO + CuO + ND nanolubricant. In addition, it is observed that the steady state is attained much earlier with the PAO + h-BN + ND nanolubricant (110 cycles) than with the PAO + CuO + ND nanolubricant (140 cycles). Taken together, ND particles have better synergy with h-BN nanoparticles than with CuO nanoparticles, and, hence, the PAO + h-BN + ND nanolubricant has better lubrication performance than PAO + CuO + ND nanolubricant.

### 3.5. Sliding of Steel–Steel Tribopair vs. Steel–Aluminium Tribopair

**Figure 18:**
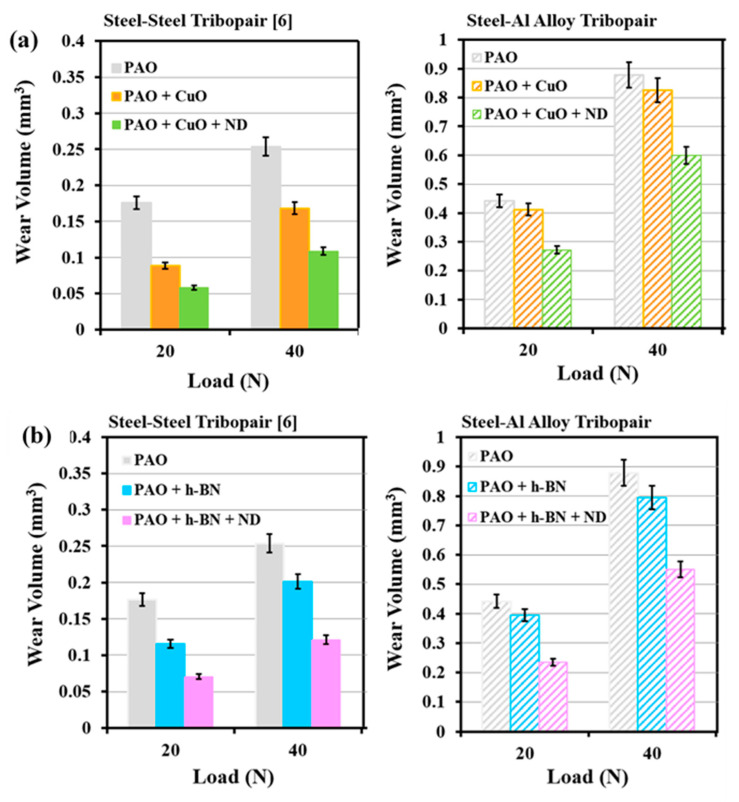
Volumetric wear of En31 steel discs [10] and Al alloy discs when AISI 52100 steel balls were slid against the discs under: under: (**a**) PAO, PAO + CuO primary additive/+ ND secondary additive lubricants and (**b**) PAO, PAO + h-BN pri-mary additive/+ ND secondary additive lubricants.

**Figure 19:**
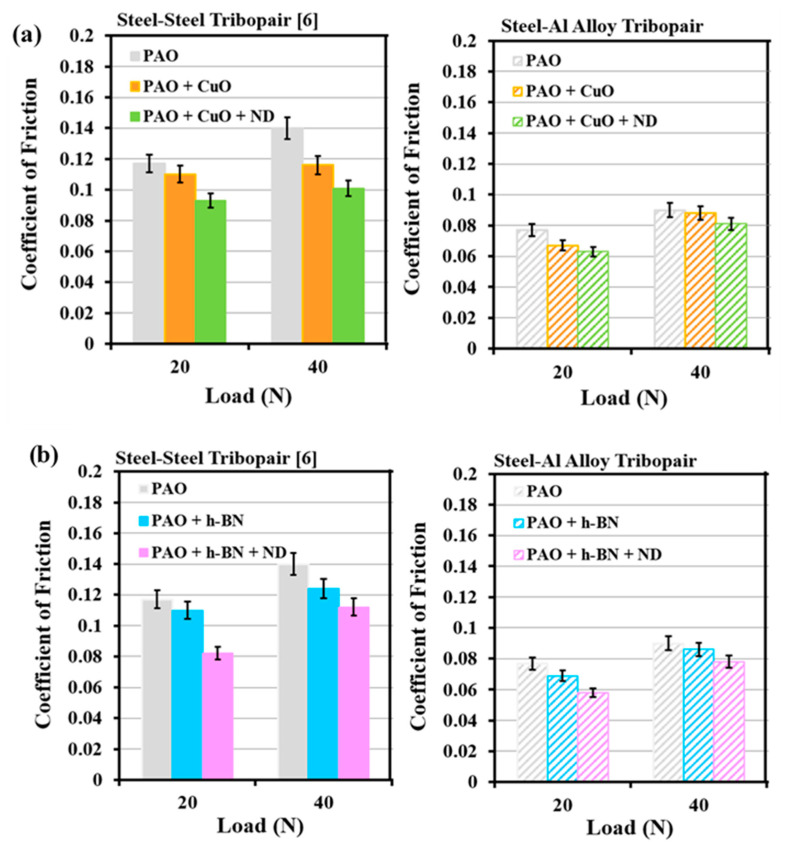
COF of steel–steel tribopair [10] and steel–aluminium tribopair when AISI 52100 steel balls were slid against the discs under: (**a**) PAO, PAO + CuO primary additive/+ ND secondary additive lubricants and (**b**) PAO, PAO + h-BN primary additive/+ ND secondary additive lubricants.

The enhancement in the lubrication performance of the steel–steel tribopair with PAO oil with CuO/h-BN nanoparticles as primary additives and ND particles as secondary additives is well-reported [10]. Similar benefits in terms of lubrication by using ND particles as the secondary additive with CuO/h-BN nanoparticles as primary additives for the steel–aluminium tribopair have been identified in the present work. However, there is a distinct difference in the magnitudes and trends of wear and friction properties between the two tribopairs, which prompts examination. Results from the present work on the steel–aluminium tribopair are compared with those on steel–steel tribopair (AISI 52100 ball slid against En31 steel disc) [10], for the same test conditions of loads (20 N and 40 N), speed (0.29 m/s), test duration (522 m) and combination of primary additives (CuO, h-BN nanoparticles, size: 60 to 90 nm) and secondary additives (ND particles, size: 60 to 90 nm). Volumetric wear (mm^3^) of En31 steel discs [10] and Al alloy discs when AISI 52100 balls were slid against the discs for all lubricant combinations are shown in Figure 18a,b. COF of the steel–steel tribopair [10] and the steel–aluminium tribopair are shown in Figure 19a,b for all lubricant combinations.

Wear volume of En31 steel discs in the presence of PAO base oil and four nanolubricants is less than those of Al alloy discs in the presence of PAO base oil and four nanolubricants, due to the higher hardness of steel discs (En31 steel: 63 HRC ≈ 830 VHN [31]) than Al alloy (AA2024: 148 VHN, measured). Based on Archard’s wear equation, specific wear rate (k) can be calculated as k = V/SN, where V is the wear volume (m^3^), S is the total sliding distance (522 m) and N is the applied normal load (N). At the load of 40 N, the specific wear rate (k) of En31 steel disc under lubrication by PAO base oil is 1.21 × 10^−14^ m^2^/N and that of Al alloy disc under lubrication by PAO base oil is 4.21 × 10^−14^ m^2^/N. Under the same sliding conditions, the rate of material loss is higher by about 3.5 times for the Al alloy disc than for the En31 disc, due to the lower hardness of the Al alloy disc compared to the En31 steel disc. Considering the energy-based approach, volume of wear is directly proportional to the energy dissipated by friction [39,41]. At the load of 40 N, the wear volume of the En31 steel disc is 0.2536 mm^3^ (Figure 18) and the energy dissipated is 2923 J, for PAO base oil lubrication. At the same load, the wear volume of the Al alloy disc is 0.8791 mm^3^ (Figure 18) and the energy dissipated is 1897 J, for PAO base oil lubrication. Comparing the wear behaviour of the discs and the energy dissipated, wear of the En31 steel disc is lower, but is associated with higher energy dissipation; vice versa for the Al alloy disc. Energy dissipated for the En31 steel disc is high, as the COF value is high (0.14, Figure 19) and it is low for Al alloy disc as the COF value is low (0.09, Figure 19), for PAO base oil lubrication. Higher energy becomes expended for the wear of harder material. A similar observation was reported earlier by Ramalho et al. [41]. With the test materials of mild steel AISI 1037, hard steel AISI 52100 and tungsten carbide with 10 wt% of cobalt (cermet, usually used for cutting tools), they observed the wear volume (m^3^) of the materials in the order: AISI 1037 > AISI 52100 > WC-Co, and the energy dissipated (J) in the order: WC-Co > AISI 52100 > AISI 1037, indicating that higher energy becomes dissipated for the wear of harder material. In the present case, this phenomenon is consistent even for the wear and energy dissipation (i.e., COF) of the En31 steel disc and the Al alloy disc with the four nanolubricants, such that under PAO + CuO, PAO + CuO + ND, PAO + h-BN and PAO + h-BN + ND lubrication, the wear volume of the En31 steel disc is lower and the COF is higher when compared to those of Al alloy discs (Figure 18 and Figure 19). The hardness of the metallic discs causes a prominent difference in the magnitudes and trends of wear and friction properties between the two tribopairs.

**Figure 20:**
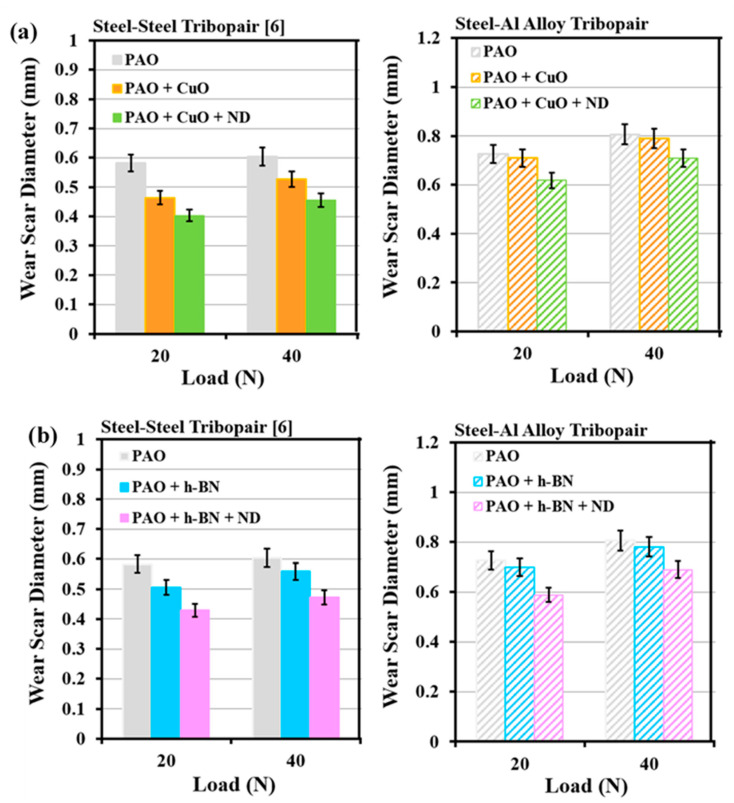
Wear scar diameter (WSD) of the AISI 52100 steel balls slid against En31 steel discs [10] and Al alloy discs under: (**a**) PAO, PAO + CuO primary additive/+ ND secondary additive lubricants and (**b**) PAO, PAO + h-BN primary additive/+ ND secondary additive lubricants.

Wear scar diameter (WSD) of the AISI 52100 steel balls slid against En31 steel discs [10] and Al alloy discs under all lubricant combinations are shown in Figure 20a,b. WSD of the AISI 52100 steel balls slid against Al alloy discs are larger than those when slid against En31 steel discs, under all lubricant combinations. To consider WSD values of the AISI 52100 steel balls as their wear value would be erroneous. Wear of the AISI 52100 steel balls slid against En31 steel discs is expected to be more than those slid against Al alloy discs, as En31 steel is harder than Al alloy. Larger values of WSD in the AISI 52100 steel balls slid against Al alloy discs are due to their larger Herztian contact area (A_H_ = π (a_H_)^2^, where A_H_ is the Hertzian contact area and a_H_ is the contact radius given by Equation (5), [42]) when a steel ball is in contact with Al alloy disc, because of the lower elastic modulus of Al alloy (75 GPa) than that of the En31 steel disc (200 GPa, [10]).

a_H_ = [(3R/2E*) (F_n_)]^1/3^, (5)

where R is the composite radius, E* is the composite elastic modulus and F_n_ is the applied normal load.

At 20 N load, the contact area in the steel–aluminium tribopair is 3.61 × 10^−8^ m^2^, and at 40 N load it is 5.74 × 10^−8^ m^2^, giving rise to mean contact pressure of 0.55 GPa and 0.70 GPa, respectively. For the steel–steel tribopair, the contact area at 20 N load is 2.36 × 10^−8^ m^2^ and at 40 N load it is 3.75 × 10^−8^ m^2^, giving rise to a mean contact pressure of 0.84 GPa and 1.06 GPa, respectively. To compare the wear of AISI 52100 steel balls for the two tribopairs in terms of weight loss poses a challenge, due to material transfer to the surfaces of the steel balls. In post-test cleaning of steel ball surfaces, prior to their weight measurement, not all the transferred material can be removed. Nevertheless, given the fact that the contact areas for the steel–steel tribopair are smaller and the contact pressures are larger than those of the steel–aluminium tribopair, it can be expected that the AISI 52100 steel balls wear more in steel–steel contact than in steel–aluminium contact. It is to be noted here that for the stainless steel–WC alloy tribopair (i.e., very hard contact), under lubrication, ND particles produce a deleterious effect of increased wear of the sliding surfaces [43]. Friction and wear are not material properties; rather, they are properties of a tribosystem. The above discussion reveals the fact that if one element of a tribosystem is replaced, the change will substantially alter the magnitudes and trends of the tribological properties of the tribosystem.

## 4. Conclusions

The role of nanodiamond (ND) particles as a secondary additive for polyalphaolefin 4 (PAO) oil lubrication of the AISI 52100 steel–AA 2024 aluminium alloy tribopair was examined. Copper oxide (CuO) and hexagonal boron nitride (h-BN) nanoparticles were taken as primary additives. The following are the main conclusions that can be drawn from the investigation:

(1) CuO and h-BN nanoparticles as primary additives to PAO base oil considerably reduce wear and COF of sliding surfaces due to the formation of tribofilms, which prevent direct metal-to-metal contact between the tribopair.
(2) ND particles as secondary additives to PAO base oil with CuO/h-BN primary additives significantly reduce the wear and COF of the sliding surfaces. As a direct consequence of the reduction in COF, due to the addition of ND particles as secondary additive, the energy loss and the power loss reduce significantly during sliding. The enhanced lubrication effect induced by ND particles is due to their (i) physicochemical and thermal properties that are beneficial for lubrication enhancement; (ii) ball bearing and polishing effects; and (iii) synergistic interaction with primary additives to form stable tribofilms.
(3) With h-BN nanoparticles as the primary additive and ND particles as the secondary additive, the PAO base oil shows the best lubrication performance. Compared to CuO, the h-BN nanoparticles are better lubrication performance-enhancers as primary additives due to their (i) inherent physicochemical and thermal properties and (ii) better synergistic interaction with ND particles. Meanwhile, ND particles increase h-BN shearing and smearing onto disc surfaces, which enhances lubrication, promotes steady state at lower sliding cycles, and comparatively reduce energy loss and power loss.
(4) Comparative understanding of the tribological performance of the steel–aluminium tribopair from the present work and that of the steel–steel tribopair from the literature reveals the difference in the magnitudes and trends of the tribological properties between the tribopairs. The comparison highlights the role of material properties of hardness and elastic modulus in influencing the characteristic performance of the sliding tribopairs, under similar lubrication conditions.
(5) Nanodiamond is a prospective material as a secondary additive to lubricant oils. In particular, the superior lubrication via synergistic interaction with primary additives makes ND particles a promising secondary lubricant additive for lubrication of hard–soft tribocontacts in engineering systems.

## Figures and Tables

**Table 1:** Rheological properties of PAO base oil [10].

Base Oil	Density g/mL. @23 °C	Dynamic Viscosity (cP)		Kinematic Viscosity (cSt)		Viscosity Index	Specific Gravity @15.6 °C	Appearance
		40 °C	100 °C	40 °C	100 °C			
PAO4	0.80	14	3	16.8	3.9	111	0.8190	clear, bright

**Table 2:** Composition of ball and disc materials (weight %).

	Al	Fe	Cu	Mn	Si	Cr	P	Mg	C
AISI 52100 (Ball)	-	95.5	-	0.25	0.15	1.3	0.03	-	0.95
Al 2024 (Disc)	93.2	0.5	4.2	0.6	0.5	0.1	-	1.6	-

**Table 3:** Estimated *h_min_* and λ values for the applied normal loads. (composite modulus of elasticity, E* = 120.94 GPa; sliding velocity, *u* = 0.29 m/s).

Normal Load (*L*)	Minimum Film Thickness (*h_min_*)	Film Thickness Parameter (λ)
10 N	10.81 nm	0.245
20 N	9.35 nm	0.212
30 N	8.58 nm	0.195
40 N	8.08 nm	0.183
50 N	7.72 nm	0.175

**Table 4:** Average percentage of elements (wt%) on Al alloy disc surfaces slid under PAO base oil and four nanolubricants, from EDS area analysis. The values are averages taken from analysis of five different area locations on each wear track of the slid Al alloy discs.

Lubricant	Elements (wt%)					
	Al	O	Cu	Mg	B	C
PAO Base Oil	87.98	8.24	2.37	1.41	-	-
PAO + CuO	59.62	20.28	19.23	0.87	-	-
PAO + CuO + ND	55.83	19.31	16.89	0.63	-	7.34
PAO + h-BN	38.80	8.33	2.47	-	50.40	-
PAO + h-BN + ND	22.80	6.21	1.31	-	51.06	18.62

**Table 5:** Energy loss (J) due to sliding under lubrication with PAO base oil and the four nanolubricants at all loads.

Lubricant	Energy Loss (J)				
	10 N	20 N	30 N	40 N	50 N
PAO Base Oil	224	803	1331	1879	2531
PAO + CuO	203	699	1299	1837	2401
PAO + h-BN	156	720	1299	1795	2270
PAO+ CuO + ND	172	657	1205	1691	2270
PAO + h-BN + ND	104	605	1143	1628	2088

**Table 6:** Power loss (mW) due to sliding under lubrication with PAO base oil and the four nanolubricants at all loads.

Lubricant	Power Loss (mW)				
	10 N	20 N	30 N	40 N	50 N
PAO Base Oil	124	447	740	1044	1406
PAO + CuO	113	389	722	1020	1334
PAO + h-BN	87	400	722	998	1261
PAO + CuO + ND	96	365	670	940	1261
PAO + h-BN + ND	58	336	635	905	1160

**Table 7:** Nanodiamond (ND) particles’ interaction with tribo-components.

Tribo-Component	ND Particles	Effect
(1) soft sliding surfaces	polish surfaces	(i) reduces friction and wear (ii) promotes uniform and conformal tribofilm formation (iii) accelerates onset of steady state
	embedment	(i) increases load-bearing capacity and hardness, reduces wear (ii) mechanically lock tribofilm onto surfaces, promotes sustained surface protection
(2) tribofilms (prevent direct metal-to-metal contact)	embedment	(i) increases load-bearing capacity and hardness, reduces wear (ii) shear soft additives, reduces friction
ND Particles feature		
(i) hard (ii) spherical shape	roll at interface (ball bearing effect)	reduces friction
(iii) high thermal conductivity	presence at interface	increases heat dissipation

## Data Availability

Data is contained within the article.
